# Transformer-Based Modeling of Directed Transfer Entropy Connectivity for EEG-Based ADHD Classification in Children

**DOI:** 10.3390/s26185786

**Published:** 2026-09-11

**Authors:** Alejandra Gomez-Rivera, Julián David Pastrana-Cortés, Andrés Marino Álvarez-Meza, Julian Gil-Gonzalez, David Cárdenas-Peña

**Affiliations:** 1 Signal Processing and Recognition Group, Universidad Nacional de Colombia, Manizales 170003, Colombia; amalvarezme@unal.edu.co; 2 Automatics Research Group, Universidad Tecnológica de Pereira (UTP), Pereira 660003, Colombia; j.pastrana@utp.edu.co (J.D.P.-C.); jugil@utp.edu.co (J.G.-G.); dcardenasp@utp.edu.co (D.C.-P.)

**Keywords:** EEG, ADHD, effective connectivity, transfer entropy, intra-subject variability, transformer networks, kernel-based learning, interpretable deep learning

## Abstract

Electroencephalography (EEG) provides a non-invasive and cost-effective tool for supporting the assessment of attention-deficit/hyperactivity disorder (ADHD). However, the nonstationary nature of EEG produces substantial variability among signal windows recorded from the same participant, which can obscure diagnostic structure and lead to inconsistent predictions. To address this problem, we propose the Contextualized Transfer Entropy Network (CTE-Net), an end-to-end deep-learning architecture that combines global content-based contextualization with nonlinear and directed EEG connectivity estimation. CTE-Net first employs a Transformer encoder to contextualize the multichannel representations within each EEG window. The resulting signals are processed using channel-wise nonlinear temporal filters and Takens delay-coordinate embeddings. A differentiable matrix-based Transfer Entropy module, formulated using Rényi’s α-entropy and a rational quadratic kernel, then estimates directed predictive information dependencies between all ordered electrode pairs. The resulting connectivity coefficients are used for ADHD-versus-control classification. The model was evaluated on a publicly available pediatric EEG dataset comprising 120 participants, equally divided between ADHD and control groups, using five fixed subject-wise folds and ten random training repetitions. At the window level, CTE-Net achieved an accuracy of 80.9±1.7%, precision of 82.7±2.1%, and sensitivity of 84.2±2.3%. At the participant level, it achieved an accuracy of 83.4% (95% CI: 78.2–88.2) and an ROC-AUC of 90.2% (95% CI: 85.1–94.6), demonstrating competitive and comparatively balanced classification performance. Beyond classification performance, the directed Transfer Entropy representation exhibited the lowest within-subject dispersion among the analyzed representation stages, with a median reduction of 38.35% relative to raw EEG. This reduction remained consistent across different PCA dimensionalities and distance definitions. These single-dataset findings support CTE-Net as a compact and interpretable methodological framework for representing directed EEG interactions while attenuating window-specific variability within individual participants.

## 1. Introduction

Attention-deficit/hyperactivity disorder (ADHD) is a common neurodevelopmental condition characterized by persistent patterns of inattention, hyperactivity, and impulsivity that frequently continue into adolescence and adulthood, with substantial effects on academic performance, social functioning, and quality of life [1]. Epidemiological studies estimate a worldwide childhood prevalence of approximately 5%, although reported rates vary across regions and diagnostic criteria [1]. Current clinical guidelines recommend integrating information from multiple sources, including parents, teachers, and clinicians, together with an assessment of symptom persistence and functional impairment [2]. Nevertheless, the diagnostic process remains partly dependent on subjective reports that may be influenced by context, comorbidities, and disagreement among observers. Moreover, no neurophysiological marker has yet demonstrated sufficient reliability and specificity to serve as a stand-alone diagnostic test for ADHD [3]. These limitations have motivated the search for objective biomarkers that can complement, rather than replace, clinical assessment.

Electroencephalography (EEG) provides a non-invasive, temporally resolved, and comparatively low-cost tool for examining large-scale neural dynamics. A broad range of spectral, event-related, and connectivity-based EEG markers have therefore been investigated in ADHD [3,4]. Among the most extensively studied spectral markers is the theta–beta ratio. Although an elevated theta–beta ratio was historically associated with ADHD, meta-analytic evidence has revealed substantial heterogeneity across participants, developmental stages, and recording protocols, limiting its reliability as a general diagnostic marker [5,6]. This has encouraged a shift from isolated spectral descriptors toward distributed network representations capable of characterizing interactions among cortical regions.

Connectivity studies have reported alterations involving frontal, parietal, and posterior regions associated with attention, executive control, and visual processing in children with ADHD [4,7,8]. However, commonly used coherence-based measures are predominantly linear and undirected, while other synchronization measures may not distinguish the source and target of an interaction. Consequently, these approaches may provide an incomplete description of nonlinear and direction-dependent relationships in large-scale EEG networks.

Transfer Entropy (TE) provides an information-theoretic framework for estimating directed predictive dependencies between dynamical processes. It quantifies the reduction in uncertainty about the future state of a target process obtained by incorporating information from the past of a source process beyond that already contained in the target’s own history [9,10]. TE can capture nonlinear and time-delayed interactions without requiring an explicit parametric model of the underlying dynamics. Nevertheless, its directional nature should not be interpreted as evidence of causal influence in the absence of the additional assumptions required for causal identification. Accordingly, TE coefficients are interpreted in this work as measures of directed predictive information rather than definitive causal relationships.

Several extensions of TE have been proposed to improve its applicability to neurophysiological data. These include bivariate and multivariate formulations for neural mass models and EEG signals [11], phase-based variants such as Phase Transfer Entropy (PTE), which focus on oscillatory phase dynamics [12], and computational frameworks such as MuTE for comparing different multivariate TE estimators [13]. More recently, kernel-based formulations have been introduced to reduce the dependence on explicit probability-density estimation and exploit the geometry encoded by positive-definite kernel matrices. De La Pava Panche et al. proposed a matrix-based TE estimator grounded in Rényi’s α-entropy, enabling data-driven estimation of directed connectivity from kernel representations [14]. This framework was subsequently extended to kernelized Phase Transfer Entropy with feature-relevance analysis for brain–computer interfaces, improving the discriminative usefulness and inspectability of EEG connectivity representations [15].

Several studies have specifically employed TE and PTE to characterize EEG connectivity in children with ADHD. Ekhlasi et al. constructed effective connectivity matrices using directed Phase Transfer Entropy across electrode pairs and frequency bands and showed that the resulting features could discriminate between children with ADHD and typically developing controls [16]. Subsequent work used PTE-derived networks to investigate group differences in connectivity strength and information-flow organization [17]. Graph-theoretic analyses further reported increased network segregation and reduced integration in ADHD, particularly in the delta and theta bands [18]. More recently, optimal multivariate TE schemes have been used to examine short- and long-range connectivity differences in the same pediatric EEG dataset [19]. Collectively, these studies support the sensitivity of TE-based representations to directional connectivity alterations in ADHD. However, they generally rely on handcrafted connectivity features and conventional classifiers, with TE computed as a fixed preprocessing operation rather than learned jointly with the classification model.

Beyond intersubject heterogeneity, an additional and comparatively underexplored challenge in EEG-based ADHD classification is within-subject variability. EEG is inherently nonstationary, and windows extracted from the same recording may differ because of fluctuations in attention, alertness, task engagement, ongoing neural activity, and measurement noise [20]. This problem is particularly relevant to ADHD, for which moment-to-moment behavioral inconsistency and response-time variability have been repeatedly documented [21,22]. Single-trial EEG studies have also identified altered neural variability in sensory responses, prestimulus activity, event-related potentials, and oscillatory dynamics, although the direction and magnitude of these effects depend on the cognitive state, task phase, and neural feature under examination [22,23,24].

From a representation-learning perspective, EEG nonstationarity can produce shifts in feature distributions and reduce the generalization ability of classification models [20,25]. In a window-based setting, these changes may appear as increased dispersion among the latent representations extracted from the same participant, potentially obscuring diagnostic structure and producing inconsistent window-level decisions. Most existing ADHD classification approaches primarily optimize separation between diagnostic groups without explicitly examining whether the learned representation becomes more compact across windows belonging to the same child. A robust representation should therefore pursue two complementary objectives: preserving diagnostically relevant differences between ADHD and control participants while attenuating nuisance fluctuations among windows from the same participant. Importantly, the geometric dispersion of learned representations should not be interpreted as a direct measure of biological neural variability. Rather, it quantifies the stability with which the model represents repeated EEG windows from an individual.

Deep-learning approaches provide an attractive alternative to handcrafted EEG descriptors by jointly learning feature representations and classification functions from raw or minimally processed signals. Convolutional neural networks and hybrid architectures have demonstrated competitive performance across several EEG-decoding applications [26,27]. Transformer-based models have extended this framework by using self-attention to contextualize latent EEG representations across an input window [28,29]. In the ADHD domain, recent convolutional and attention-based architectures have reported promising classification performance from raw EEG signals [30,31]. However, these models generally treat EEG as a generic multichannel time series or encode connectivity through undirected similarity measures. Consequently, they do not explicitly represent nonlinear and directed information transfer between electrode signals.

A complementary line of research has integrated connectivity estimation into differentiable EEG architectures. The Takens-based Kernel Transfer Entropy Connectivity Network (TEKTE-Net) was introduced for motor-imagery classification by combining channel-wise nonlinear temporal filtering, delay-coordinate state-space reconstruction, and matrix-based TE estimation within a unified learning pipeline [32]. TEKTE-Net learns temporal signal representations and directed predictive dependencies jointly with the classification function, producing inspectable connectivity patterns associated with model decisions. Nevertheless, the architecture has so far been investigated in motor-imagery BCIs and has not been adapted to ADHD classification or combined with global self attention-based contextualization.

To address these limitations, this work introduces the Contextualized Transfer Entropy Network (CTE-Net), an end-to-end architecture that combines global content-based contextualization with differentiable kernel Transfer Entropy estimation for EEG-based ADHD classification. Unlike approaches in which connectivity is computed as a fixed preprocessing step, CTE-Net jointly learns contextualized signal representations, nonlinear temporal features, directed-connectivity coefficients, and the final classification function.

CTE-Net first employs a Transformer encoder to establish content-dependent interactions among multichannel EEG representations across the complete input window. The Transformer is therefore used as a global contextualization mechanism, while temporal order and delayed dynamics are explicitly represented in the subsequent channel-wise temporal-filtering and Takens state-space reconstruction stages. A matrix-based Transfer Entropy module, formulated using Rényi’s α-entropy and a rational quadratic kernel, then estimates nonlinear, time-delayed, and directed predictive dependencies for all ordered electrode pairs. After excluding self-connections, the resulting coefficients form a compact directed-connectivity representation for ADHD-versus-control classification.

The relevance of CTE-Net extends beyond classification performance. By transforming contextualized EEG activity into directed information-flow representations, the architecture is intended to preserve diagnostically relevant interchannel relationships while attenuating window-specific fluctuations that contribute to within-subject dispersion. This hypothesis is evaluated by tracking the organization of EEG windows across the raw input, Transformer-contextualized representation, nonlinear temporal-filtering stage, and final TE connectivity space. The proposed model was evaluated on a publicly available pediatric EEG dataset comprising 120 participants using five fixed subject-wise folds and ten random training repetitions. CTE-Net achieved competitive classification performance relative to established deep-learning baselines. More importantly, its directed TE representation exhibited the lowest within-subject dispersion among the analyzed stages, with a median reduction of 38.35% relative to raw EEG. The direction of this effect remained consistent across alternative dimensionalities and distance definitions.

The main contributions of this work are as follows: (i) an end-to-end architecture that combines global self-attention-based contextualization, channel-wise nonlinear temporal filtering, Takens state-space reconstruction, and differentiable kernel Transfer Entropy to learn explicit directed EEG connectivity representations; (ii) a multi-stage analysis of within-subject representation dispersion that quantifies how windows belonging to the same participant are reorganized from the raw EEG domain to the final directed-connectivity space, including robustness analyses across alternative dimensionalities and distance definitions; and (iii) a subject-independent experimental framework based on fixed stratified group partitions, ten random training repetitions, component-wise ablation, paired statistical comparisons with deep-learning baselines, latent-space analyses, and subject-specific inspection of learned channel-indexed directed-connectivity patterns.

The remainder of this paper is organized as follows: Section 2 describes the EEG dataset, preprocessing pipeline, proposed architecture, training strategy, and subject-independent evaluation protocol. Section 3 reports and discusses the experimental results, including hyperparameter tuning, interpretability analysis, ablation study, comparative performance evaluation, and statistical significance tests. Finally, Section 4 presents the concluding remarks and future research directions.

## 2. Materials and Methods

In this work, we extend the kernel-based TE paradigm to pediatric ADHD classification by proposing CTE-Net, an end-to-end architecture that integrates Transformer-based contextualization with differentiable directed connectivity estimation. The model first processes each multichannel EEG window using a Transformer encoder to obtain globally contextualized channel representations. These representations are then processed through channel-wise nonlinear temporal filters and Takens delay-coordinate embeddings. A differentiable kernel-based Transfer Entropy module estimates directed predictive information dependencies for all ordered electrode pairs, and the resulting connectivity representation is used for binary classification. In this manner, CTE-Net combines global contextual modeling with nonlinear, time-delayed, and directed EEG connectivity analysis. Table 1 summarizes the main characteristics of the cohort, EEG recording, and protocol considered in the present study.

### 2.1. Dataset and Preprocessing

The experimental evaluation was conducted using the publicly available “EEG data for ADHD/control children” database distributed through IEEE DataPort by Nasrabadi et al. [33]. The dataset contains EEG recordings from 121 children between 7 and 12 years of age, including participants diagnosed with Attention-Deficit/Hyperactivity Disorder (ADHD) and typically developing controls [17,34]. ADHD diagnoses were established by an experienced child psychiatrist according to the DSM-IV criteria. At the time of data acquisition, the children in the ADHD group had received methylphenidate treatment for no longer than six months, while the control participants had no reported history of psychiatric or neurological disorders  [17,34].

During EEG acquisition, the participants performed a visual-attention task based on cartoon scenes. Each image contained between 5 and 16 characters, and the children were instructed to count the number of figures displayed in the scene. After a response was provided, the next image was immediately presented. Therefore, the total duration of each recording depended on the response time of the participant  [34,35]. This experimental paradigm was designed to engage sustained visual-attention processes, which are frequently affected in children with ADHD.

**Figure 1:**
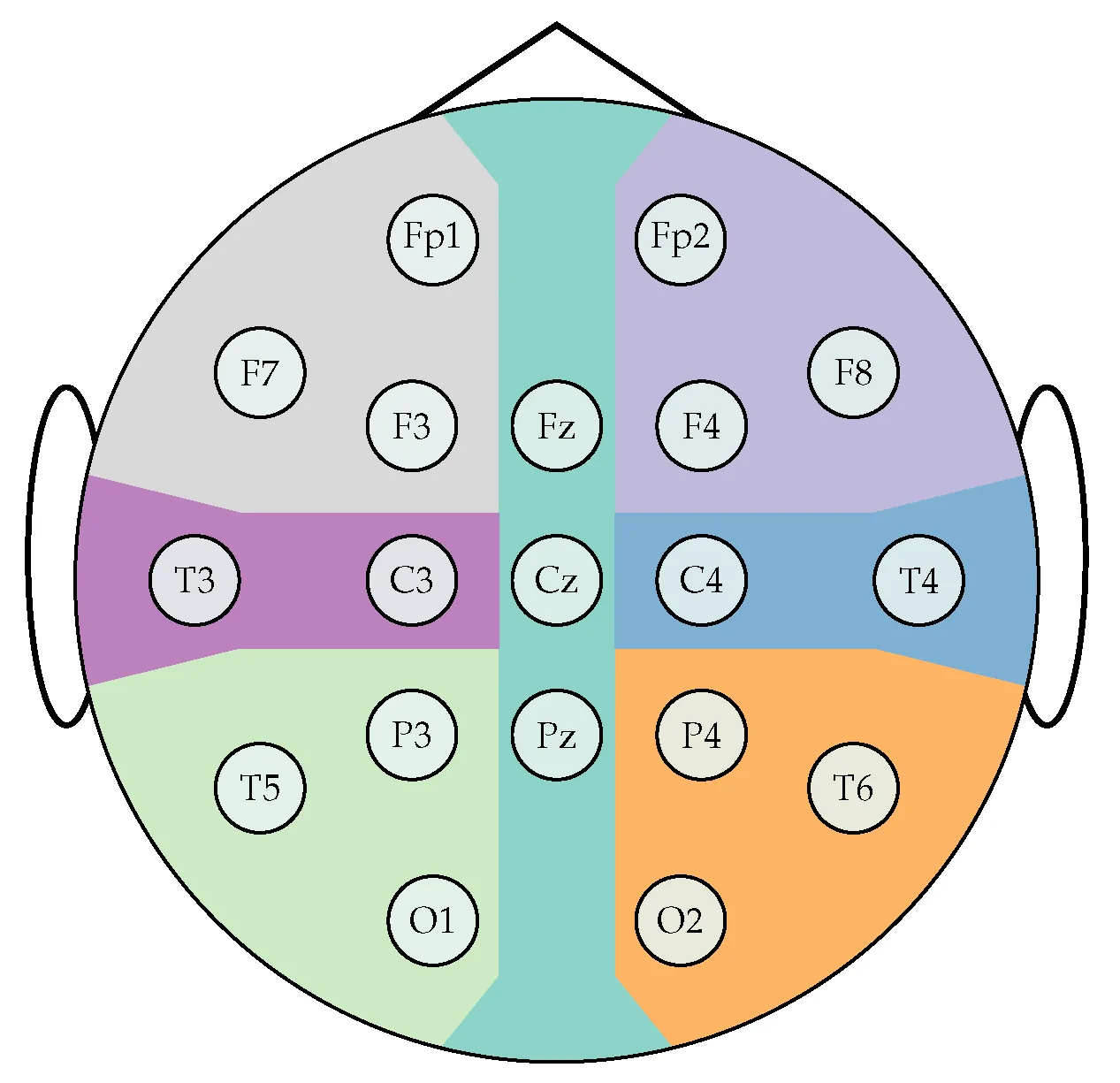
Scalp electrode montage following the international 10–20 system for the ADHD/control children dataset. The 19 EEG channels are grouped into anatomically defined cortical regions, indicated by color: ■ frontal left and central left, ■ midline electrodes (Fz, Cz, Pz), ■ frontal right, ■ central right, ■ centro-parietal and parietal left, ■ centro-parietal and parietal right, and ■ occipital region.

EEG activity was acquired at a sampling frequency of 128 Hz using 19 scalp electrodes positioned according to the international 10–20 system: Fp1, Fp2, F3, F4, F7, F8, Fz, C3, C4, Cz, P3, P4, Pz, T3/T7, T4/T8, T5/P7, T6/P8, O1, and O2. The spatial distribution of the electrodes is illustrated in Figure 1. The recordings were referenced during acquisition using the A1 and A2 earlobe electrodes. This database has previously been employed to investigate altered EEG interactions in ADHD using directed connectivity approaches, including Phase Transfer Entropy, directed Phase Transfer Entropy, and multivariate Transfer Entropy [16,19].

The original dataset comprised 61 participants with ADHD and 60 controls. To define a balanced cohort, one ADHD participant was randomly selected and excluded before window segmentation, fold construction, hyperparameter optimization, model training, and evaluation. The selection was not based on EEG signal characteristics or quality, demographic or clinical information, model predictions, or classification performance. The resulting balanced cohort comprised 120 children, with 60 participants in the ADHD group and 60 in the control group, and was maintained across all models and experimental analyses. The EEG recordings were loaded in their original temporal form without applying additional artifact rejection, independent component analysis, frequency filtering, or spectral-band decomposition. This strategy was adopted to minimize preprocessing assumptions and allow the proposed network to learn temporal and connectivity-related patterns directly from the available multichannel recordings. As a post hoc signal-quality assessment, EEG windows were screened for nonfinite values, flat channels, extreme amplitude, kurtosis, frontal low- and high-frequency activity, and 50-Hz line noise. Participant-balanced median/MAD thresholds were estimated from 30 windows per participant, with robust z>5 defining potentially contaminated windows. Without retraining, out-of-fold metrics were recalculated after excluding flagged windows and reaggregating participant probabilities; z>4 and z>6 were examined as sensitivity thresholds.

Each continuous recording was partitioned into 4-s windows, corresponding to 512 temporal samples at the original sampling frequency of 128 Hz. Consecutive windows were extracted with 50% overlap; therefore, adjacent segments were separated by 256 samples, equivalent to a temporal shift of 2 s. The *n*-th EEG segment was represented as

(1) $$X^{\left(n\right)}\in R^{C\times T},$$

where C=19 is the number of EEG channels and T=512 is the number of temporal samples per window. Each segment retained the diagnostic label and subject identifier associated with its original recording.

The 50% overlap increased temporal coverage and provided additional but partially redundant segments during model optimization; therefore, adjacent windows were not treated as independent statistical observations. Subject-wise partitioning kept all windows from each participant within a single training, validation, or test subset, preventing information leakage. Primary inference used one aggregated out-of-fold prediction per participant and participant-level bootstrap confidence intervals, while window-level metrics were interpreted as descriptive.

The number of windows per participant ranged from 30 to 167, with a median of 64 and an interquartile range of 51.75–77, yielding 8213 windows in total. Participants with ADHD contributed 4556 windows, with a median of 70.5 (IQR: 53.75–91.5), whereas controls contributed 3657 windows, with a median of 59.5 (IQR: 51–68). Because training and window-level evaluation operated on individual windows, participants with longer recordings contributed more observations. Nevertheless, the primary participant-level evaluation assigned one aggregated out-of-fold prediction to each participant, giving all participants equal weight in the reported participant-level metrics.

Subject identifiers were preserved throughout the experimental pipeline and subsequently used to construct the cross-validation partitions. Thus, all windows belonging to a given participant were assigned exclusively to a single training, validation, or test subset, preventing subject-level information leakage during model optimization and evaluation.

### 2.2. CTE-Net Architecture

#### 2.2.1. Transformer-Based Contextualization

Let a multichannel EEG window be represented as $X\in R^{C\times T}$, where *C* and *T* denote the number of channels and temporal samples, respectively. Each temporal sample is treated as a sequence token containing the simultaneous activity of all EEG channels. The normalized token sequence is projected onto a *d*-dimensional latent space as:

(2) $$\text{E}={\mathrm{LN}}_{C}\;\left({\text{X}}^{⊤}\right){\text{W}}_{e}+{\text{b}}_{e},\;\text{E}\in R^{T\times d}.$$

A stack of *L* standard Transformer encoder blocks [28], comprising multi-head self-attention, feed-forward layers, residual connections, and layer normalization, produces

(3) $$\text{H}={\mathrm{TrEnc}}_{L}\left(\text{E}\right)\in R^{T\times d}.$$

The contextualized representation is projected back onto the original channel space:

(4) $${\text{X}}_{f}={\left[\mathrm{LN}\left(\text{H}\right){\text{W}}_{p}+{\text{b}}_{p}\right]}^{⊤}\in R^{C\times T}.$$

Therefore, each row of ${\text{X}}_{f}$ represents an EEG channel contextualized using information from the complete temporal sequence and the remaining channels. The Transformer configuration is reported in Table 2.

No explicit positional encoding was incorporated into the Transformer encoder. Consequently, the self-attention mechanism does not, by itself, encode absolute temporal order or temporal distance based on token position. In CTE-Net, self-attention is instead used as a content-based global contextualization mechanism, allowing each temporal sample to be represented in relation to the multichannel activity observed across the entire EEG window. The resulting contextualized sequence remains indexed along the original time axis and is subsequently processed through channel-wise depthwise temporal convolution and Takens delay-coordinate reconstruction. These stages explicitly exploit temporal ordering and construct past and present states according to the reconstruction delay τ and the source–target interaction lag μ. Thus, global content-based contextualization and the explicit modeling of ordered and delayed temporal dependencies are assigned to distinct components of the architecture.

#### 2.2.2. Differentiable Directed Transfer Entropy Estimation

The Transformer-contextualized signals are independently processed through channel-wise nonlinear temporal filters. The complete filtering operation is expressed as

(5) $$\text{Φ}=\mathrm{BN}\left[\mathrm{AvgPool}\left(\mathrm{ReLU}\left(DWConv1D(X_{f})\right)\right)\right]\in R^{C\times T_{\phi }},$$

where DWConv1D denotes a depthwise temporal convolution, so that each contextualized channel is filtered independently. The kernel size, stride, and pooling configuration are provided in Table 2.

For each ordered source–target pair $\left(c,c^{\prime }\right)$, delay-coordinate embeddings are constructed from the filtered signals [36]. At temporal anchor *t*, the source past, target past, and target present states are defined as:

(6) $$\begin{array}{cc} {\text{z}}_{x_{c}^{-}}\left(t\right)\; & ={\left[\phi_{c}\left(t-\mu -j\tau \right)\right]}_{j=0}^{D_{x}-1}\in R^{D_{x}}, \end{array}$$

(7) $$\begin{array}{cc} {\text{z}}_{y_{c^{\prime }}^{-}}\left(t\right)\; & ={\left[\phi_{c^{\prime }}\left(t-j\tau \right)\right]}_{j=1}^{D_{y}}\in R^{D_{y}}, \end{array}$$

(8) $$\begin{array}{cc} z_{y_{c^{\prime }}}\left(t\right)\; & =\phi_{c^{\prime }}\left(t\right). \end{array}$$

Here, $D_{x}$ and $D_{y}$ are the source and target embedding dimensions, τ is the reconstruction delay, and μ represents the delayed source–target interaction.

Collecting the states over all valid temporal anchors yields ${\text{Z}}_{x_{c}^{-}}$, ${\text{Z}}_{y_{c^{\prime }}^{-}}$, and ${\text{Z}}_{y_{c^{\prime }}}$. Before kernel evaluation, trainable bias-free linear projections are applied:

(9) $${\tilde{\text{Z}}}_{s}={\text{Z}}_{s}{\text{W}}_{s},\;s\in \left\{x_{c}^{-},y_{c^{\prime }}^{-},y_{c^{\prime }}\right\}.$$

For each projected state representation, a Gram matrix is constructed using the rational quadratic kernel [37]. Specifically, for $s\in \{x_{c}^{-},y_{c^{\prime }}^{-},y_{c^{\prime }}\}$,

(10) $${\left[{\text{K}}_{s}\right]}_{ij}=\kappa_{\mathrm{RQ}}\left({\tilde{\text{z}}}_{s,i},{\tilde{\text{z}}}_{s,j}\right)=a^{2}{\left(1+\frac{{∥{\tilde{\text{z}}}_{s,i}-{\tilde{\text{z}}}_{s,j}∥}_{2}^{2}}{2\alpha_{\mathrm{RQ}}\ell^{2}}\right)}^{-\alpha_{\mathrm{RQ}}},$$

where *a*, *ℓ*, and $\alpha_{\mathrm{RQ}}$ denote the kernel amplitude, length scale, and scale-mixture parameter, respectively. The resulting matrices ${\text{K}}_{x_{c}^{-}}$, ${\text{K}}_{y_{c^{\prime }}^{-}}$, and ${\text{K}}_{y_{c^{\prime }}}$ encode pairwise similarities between the reconstructed states at the valid temporal anchors. The kernel configuration is reported in Table 2. For positive kernel parameters, the Rational Quadratic kernel produces positive-semidefinite Gram matrices. Moreover, the Hadamard products used to construct the joint kernel representations preserve positive semidefiniteness.

Numerical stability was ensured by enforcing strictly positive kernel parameters and using $\varepsilon =10^{-8}$ as a lower bound for the kernel length scale *ℓ* and shape parameter $\alpha_{\mathrm{RQ}}$, as well as a stabilizing constant during trace normalization and logarithmic entropy evaluation.

For a positive semidefinite matrix A, let the trace-normalization operator be defined as:

(11) $$N\left(\text{A}\right)=\frac{\text{A}}{\mathrm{tr}(\text{A})}.$$

The matrix-based Rényi entropy of order $\alpha_{R}$ is then defined as:

(12) $$H_{\alpha_{R}}\left(\text{A}\right)=\frac{1}{1-\alpha_{R}}\log\left[\mathrm{tr}\left(N{\left(\text{A}\right)}^{\alpha_{R}}\right)\right].$$

This matrix-based entropy functional follows the positive definite kernel formulation introduced for information-theoretic learning and subsequently used for kernel-based Transfer Entropy estimation [14,38].

For multiple variables represented by Gram matrices ${\text{K}}_{1},\ldots ,{\text{K}}_{M}$, the corresponding joint entropy is computed from their Hadamard product:

(13) $$H_{\alpha_{R}}\left({\text{K}}_{1},\ldots ,{\text{K}}_{M}\right)=H_{\alpha_{R}}\left({\text{K}}_{1}⊙\cdots ⊙{\text{K}}_{M}\right),$$

where ⊙ denotes the Hadamard product. The trace normalization included in $H_{\alpha_{R}}\left(\;\cdot \;\right)$ ensures that both marginal and joint kernel representations have unit trace before evaluating the entropy.

Accordingly, the directed Transfer Entropy from source channel *c* to target channel $c^{\prime }$ is computed as

(14) $$\begin{array}{cc} TE_{c\to c^{\prime }}= & H_{\alpha_{R}}\left({\text{K}}_{y_{c^{\prime }}^{-}},{\text{K}}_{x_{c}^{-}}\right)-H_{\alpha_{R}}\left({\text{K}}_{y_{c^{\prime }}},{\text{K}}_{y_{c^{\prime }}^{-}},{\text{K}}_{x_{c}^{-}}\right)\\ \; & +H_{\alpha_{R}}\left({\text{K}}_{y_{c^{\prime }}},{\text{K}}_{y_{c^{\prime }}^{-}}\right)-H_{\alpha_{R}}\left({\text{K}}_{y_{c^{\prime }}^{-}}\right). \end{array}$$

Repeating this computation over all ordered channel pairs produces the directed connectivity matrix

(15) $$\text{TE}={\left[TE_{c\to c^{\prime }}\right]}_{c,c^{\prime }=1}^{C}\in R^{C\times C}.$$

Since the source and target states have different roles in the formulation, TE is generally asymmetric. Therefore, its coefficients represent directed predictive information dependencies between Transformer-contextualized EEG channels rather than undirected similarity relationships. Although theoretical Transfer Entropy is non-negative, the present matrix-based formulation is a finite-sample estimator and does not explicitly enforce non-negativity. Consequently, negative estimates may occur and should not be interpreted as negative information transfer. Furthermore, because the Transformer, temporal filters, and linear projections preceding the TE module are optimized end-to-end using the supervised classification objective, the resulting coefficients quantify directed predictive dependencies within the learned representation space rather than conventional Transfer Entropy estimated directly from the raw EEG. Accordingly, a labeled coefficient such as F8→C4 denotes a directed dependency between channel-indexed coordinates of the learned contextualized representation and should not be interpreted as conventional Transfer Entropy between the original F8 and C4 electrode signals or as a source-level cortical interaction.

#### 2.2.3. Connectivity-Based Classification

The diagonal coefficients of TE represent self-connections and are excluded from the classification representation. The remaining directed coefficients are vectorized as

(16) $${\text{h}}_{\mathrm{TE}}=\mathrm{vec}\left(\left\{TE_{c\to c^{\prime }}|c\neq c^{\prime }\right\}\right)\in R^{C(C-1)}.$$

The resulting connectivity vector is processed by a fully connected classification head, and the estimated probability of ADHD is given by

(17) $$\hat{y}=\sigma \left[w_{o}^{⊤}{\mathrm{Dropout}}_{p_{d}}\left(\mathrm{ReLU}\left(W_{h}h_{\mathrm{TE}}+b_{h}\right)\right)+b_{o}\right],$$

where σ(·) denotes the sigmoid function and $p_{d}$ is the dropout probability. The complete architecture is optimized end-to-end using binary cross-entropy evaluated directly from the classification logits.

### 2.3. Training Strategy and Evaluation Protocol

CTE-Net was evaluated using five-fold stratified group cross-validation, with each participant treated as an independent group. At each iteration, 24 participants were held out for testing, while the remaining participants were divided into subject-independent training and validation subsets. Thus, no participant contributed windows to more than one subset, and each participant appeared once in the test set.

The model was trained end-to-end at the window level using binary cross-entropy. A single hyperparameter configuration was selected through 20 Optuna trials [39], using the mean validation accuracy across the five predefined subject-wise folds as the optimization objective. Within each fold, training, validation, and test participants were mutually exclusive, and only the corresponding validation subset guided early stopping and checkpoint selection. No test-set performance metric was included in hyperparameter optimization. The selected configuration, reported in Table 2, was then fixed for all five folds and all ten random training repetitions. Standardization and PCA were restricted to the post hoc representation analysis and did not influence model training, hyperparameter selection, or classification predictions.

Within the Transfer Entropy module, $D_{x}$, $D_{y}$, τ, and μ were tuned through the 20-trial Optuna study over the ranges $D_{x},D_{y}\in \left[1,10\right]$, τ∈[1,5], and μ∈[0,10]. The selected values were $D_{x}=6$, $D_{y}=1$, τ=2, and μ=2. The Rényi order $\alpha_{R}=2$ and the Rational Quadratic kernel parameters $a=\ell =\alpha_{\mathrm{RQ}}=1$ were fixed, whereas the linear projections preceding kernel evaluation were learned end-to-end.

A post hoc joint sensitivity analysis was conducted for $D_{x}$, $D_{y}$, τ, and μ, as these parameters directly determine the temporal organization of the reconstructed states and have shown relevant sensitivity in related Takens-based kernel Transfer Entropy architectures [32]. The originally selected configuration and ten feasible alternatives were evaluated across the five predefined subject-wise folds to characterize their combined sensitivity.

The complete five-fold procedure was repeated using ten random seeds and identical subject partitions, resulting in 50 trained models. The final CTE-Net configuration contained 128,578 trainable parameters, corresponding to approximately 0.49 MiB of parameter storage in FP32. All experiments were conducted on the same workstation equipped with an NVIDIA Quadro RTX 5000 GPU, an eight-core CPU with 16 processing threads, and 16 GB of system RAM. The available execution records indicated approximate training times of 5–7 min per fold and 25–35 min for one complete five-fold repetition, while the complete 20-trial Optuna study required approximately 8.1 h. For each EEG window, the Transfer Entropy module evaluated all C(C−1)=342 ordered electrode pairs, excluding self-connections. ADHD was considered the positive class, and window-level predictions were obtained using a probability threshold of 0.5. Performance was quantified as

(18) $$\begin{array}{cccccc} \mathrm{Accuracy}\; & =\frac{\mathrm{TP}+\mathrm{TN}}{\mathrm{TP}+\mathrm{TN}+\mathrm{FP}+\mathrm{FN}},\; & \mathrm{Precision}\; & =\frac{\mathrm{TP}}{\mathrm{TP}+\mathrm{FP}},\; & \mathrm{Sensitivity}\; & =\frac{\mathrm{TP}}{\mathrm{TP}+\mathrm{FN}}. \end{array}$$

For each seed, metrics were averaged across the five test folds and reported as the mean and standard deviation across the ten seeds. CTE-Net and the baselines were compared using paired two-sided Wilcoxon signed-rank tests with Holm correction [40].

These seed-based comparisons were retained as an exploratory assessment of the computational variability associated with repeated model training. The random seeds were not interpreted as independent clinical observations. Participant-level performance and uncertainty were therefore evaluated separately using the participants as the sampling units, as described below.

For the participant-level evaluation, the out-of-fold ADHD probabilities assigned to all windows belonging to participant *s* under random training seed *r* were averaged as

(19) $${\bar{p}}_{s}^{\left(r\right)}=\frac{1}{N_{s}}\sum_{i=1}^{N_{s}}p_{s,i}^{\left(r\right)}$$

where $N_{s}$ is the number of windows available for participant *s* and $p_{s,i}^{\left(r\right)}$ is the ADHD probability assigned to window *i*. A participant was classified as ADHD when ${\bar{p}}_{s}^{\left(r\right)}\geq 0.5$ and as control otherwise. Accuracy, sensitivity, specificity, precision, and F1-score were calculated from the resulting participant-level decisions, whereas ROC-AUC was calculated directly from the continuous participant-level probabilities.

The participant-level metrics were calculated separately for each of the ten random training seeds using the 120 out-of-fold participants. Point estimates were subsequently obtained by averaging the corresponding metric across the ten seeds. The seeds were regarded as repeated model fits representing optimization variability and were not treated as independent clinical observations. Ninety-five percent confidence intervals were estimated using 5000 stratified percentile bootstrap resamples at the participant level [41]. Within each bootstrap replicate, control and ADHD participants were resampled separately with replacement, the same resampled participant indices were applied across all ten seeds, and the resulting seed-specific metrics were averaged.

All baseline architectures were trained and evaluated under the same experimental protocol as CTE-Net, using identical training, validation, and test participant assignments, input windows, raw EEG preprocessing, hyperparameter-tuning budget of 20 Optuna trials, validation-based early-stopping and checkpoint-selection criteria, and ten random training repetitions per fold. Although the hyperparameter search spaces were architecture-specific, the same optimization budget and validation objective were applied to every model.

Both ablation variants were evaluated using the same fixed subject-wise folds and ten random seeds as CTE-Net. Seed-level differences were assessed using paired Wilcoxon signed-rank tests with Holm correction across six comparisons. For the participant-level analysis, one consensus decision per participant and model was obtained by averaging the out-of-fold probabilities across seeds and applying a threshold of 0.5. Exact McNemar tests [42] were then performed with Holm correction across the two ablation comparisons.

Sensitivity of predictive performance and learned connectivity to preprocessing and reference choice was assessed by retraining CTE-Net using frequency-filtered and quality-screened EEG or common average reference. Both analyses used the fixed configuration, five subject-wise folds, and ten seeds without additional hyperparameter optimization. Complete methods and results are provided in Supplementary Data S4.

### 2.4. Representation Analysis

The original EEG, Transformer output $X_{f}$, temporal-filter output Φ, and Transfer Entropy representation were extracted exclusively from out-of-fold test windows. Each representation was vectorized, standardized within each seed–fold execution, and projected onto 30 principal components for the main analysis.

Let ${\text{z}}_{s,i}^{\left(\ell ,r\right)}$ denote the representation of window *i* from participant *s*, at stage *ℓ* and repetition *r*. Within-subject dispersion and its change relative to the original EEG were computed as:

(20) $$\begin{array}{cccc} \mu_{s}^{\left(\ell ,r\right)} & =\frac{1}{N_{s}}\sum_{i=1}^{N_{s}}z_{s,i}^{\left(\ell ,r\right)},\; & D_{s}^{\left(\ell ,r\right)} & =\frac{1}{N_{s}}\sum_{i=1}^{N_{s}}{∥z_{s,i}^{\left(\ell ,r\right)}-\mu_{s}^{\left(\ell ,r\right)}∥}_{2},\\ {\tilde{D}}_{s}^{\left(\ell ,r\right)} & =\frac{D_{s}^{\left(\ell ,r\right)}}{G^{\left(\ell ,r\right)}},\; & \Delta_{s}^{\left(\ell \right)} & =100\left(\frac{{\tilde{D}}_{s}^{\left(\ell \right)}}{{\tilde{D}}_{s}^{\left(\mathrm{EEG}\right)}}-1\right), \end{array}$$

where $N_{s}$ is the number of windows and $G^{\left(\ell ,r\right)}$ is the mean pairwise Euclidean distance among at most 1200 test windows in the corresponding representation space. Participant-level dispersion was averaged across seeds before statistical analysis. Lower ${\tilde{D}}_{s}^{\left(\ell ,r\right)}$ values indicate more compact within-subject representations, whereas negative $\Delta_{s}^{\left(\ell \right)}$ values indicate a reduction relative to raw EEG. These quantities describe representation-space dispersion rather than physiological EEG variability.

Diagnostic organization was evaluated using the ADHD–control silhouette coefficient at the window level, the diagnostic silhouette of participant centroids [43], and the ratio $R={\bar{d}}_{\mathrm{same}}/{\bar{d}}_{\mathrm{different}}$ between same-class and different-class centroid distances. Higher silhouette values and lower ratios indicate better diagnostic organization.

Finally, TE stability was evaluated by comparing each window-level connectivity vector with the mean of the remaining windows from the same participant. Spearman correlation measured the stability of the global connection ranking, while Jaccard similarity measured the overlap between the strongest 10% of directed connections. Both measures were averaged across windows and seeds to obtain one value per participant.

Participant-level dispersion values were averaged across seeds and compared among stages using the Friedman test, followed by paired Wilcoxon signed-rank tests with Holm correction. Effect sizes were quantified using matched rank-biserial correlations, and 95% confidence intervals were estimated from 5000 participant-level bootstrap resamples. Robustness was assessed by repeating the analysis with 15, 30, and 60 PCA components and using pairwise cosine distance without PCA. Diagnostic-organization metrics were averaged across folds within each seed and analyzed using the same paired procedure.

For each representation stage, the standardization mean and standard deviation and the PCA basis were computed independently from the pooled out-of-fold test windows of that stage and seed-fold, prior to projection onto the leading 30 components. Because this fitting is representation-specific, we repeated the dispersion analysis using pairwise cosine distance on the standardized features without PCA, confirming that the reduction in within-subject dispersion is not an artifact of the per-representation standardization or PCA basis.

## 3. Results and Discussion

CTE-Net was evaluated under the subject-independent protocol described in Section 2.3. The results examine four complementary aspects of the proposed architecture: classification performance and statistical comparison, model robustness and sensitivity, the organization and stability of the learned representation space, and subject-specific directed-connectivity patterns associated with correct and incorrect classification decisions.

### 3.1. Classification Performance and Statistical Comparison

Classification performance was examined from two complementary statistical perspectives. First, the seed-level analysis characterized the variability associated with repeated model optimization under the same fixed subject-wise partitions. Mean and standard deviation across the ten random seeds were reported, and paired Wilcoxon signed-rank tests with Holm correction were used to assess whether the observed performance differences were consistent across repeated model fits. Because the subject partitions were reused, this analysis was interpreted as an assessment of optimization stability and not as inference based on independent clinical observations. Second, participant-level performance was evaluated using out-of-fold predictions, participant-level bootstrap confidence intervals, and exact paired McNemar tests, thereby treating the participant as the primary unit for evaluating generalization.

Table 3 presents the complete window-level classification performance of CTE-Net and the baseline models. In addition to accuracy, precision, and sensitivity, specificity, balanced accuracy, F1-score, and ROC-AUC are reported to provide a more complete assessment of class-specific behavior. These complementary metrics are particularly relevant for identifying models whose performance may be driven by a tendency to favor the ADHD class.

The window-level results reveal distinct classification profiles among the evaluated models. ShallowConvNet achieved the strongest overall point-estimate profile, leading in accuracy, balanced accuracy, specificity, precision, F1-score, and ROC-AUC, although it exhibited lower sensitivity than CTE-Net. CTE-Net and EEGNet showed similarly competitive and comparatively balanced performance across the evaluated metrics, indicating that their classification results were not predominantly driven by either diagnostic class. In contrast, T-GARNet exhibited higher sensitivity but lower specificity and precision, suggesting a greater tendency to classify windows as ADHD. This behavior was even more pronounced for MultiStream, whose high sensitivity was accompanied by markedly lower specificity, balanced accuracy, precision, and ROC-AUC. IMC-BGT also showed a less balanced performance profile. Overall, these results demonstrate why sensitivity should be interpreted jointly with specificity, balanced accuracy, F1-score, and ROC-AUC when comparing the models.

The row-normalized window-level confusion matrices for all evaluated models are provided in Supplementary Data S1. These matrices complement the reported class-specific metrics and facilitate inspection of each model’s tendency to classify windows as ADHD or control.

To complement this descriptive performance analysis, the consistency of the differences between CTE-Net and the baseline models across repeated training runs was examined using paired Wilcoxon signed-rank tests with Holm correction. These exploratory seed-level comparisons are reported in Table 4.

Within the exploratory seed-level analysis, CTE-Net and EEGNet showed comparable accuracy, precision, and sensitivity across repeated model fits. ShallowConvNet achieved higher point estimates for accuracy and precision; however, only the precision difference remained significant after Holm correction. CTE-Net showed consistently higher accuracy and precision than T-GARNet, IMC-BGT, and MultiStream. For sensitivity, CTE-Net significantly outperformed only IMC-BGT, whereas no significant differences were found relative to EEGNet, ShallowConvNet, T-GARNet, or MultiStream. Overall, these results indicate that CTE-Net provides a stable balance among accuracy, precision, and sensitivity across repeated model fits.

Because ADHD diagnosis and the intended generalization unit correspond to the participant rather than to individual EEG windows, performance was additionally evaluated after aggregating the out-of-fold window probabilities within each participant. Table 5 reports the resulting participant-level point estimates and 95% confidence intervals for all evaluated models. The point estimates summarize performance across the ten repeated model fits, whereas the confidence intervals were obtained by resampling participants and therefore quantify uncertainty with respect to the study cohort.

**Table 4:** Exploratory seed-level comparisons between CTE-Net and the baseline models. Results are reported as the mean and standard deviation across ten random training seeds. Paired Wilcoxon signed-rank tests were used to evaluate the consistency of the differences across repeated model fits, with Holm correction applied jointly across the 15 comparisons. Random seeds were treated as computational repetitions and not as independent clinical observations.

Model	Accuracy (%)			Precision (%)			Sensitivity (%)		
	Mean ± SD	$p_{\text{Holm}}$	vs.	Mean ± SD	$p_{\text{Holm}}$	vs.	Mean ± SD	$p_{\text{Holm}}$	vs.
**CTE-Net**	**80.9** ± **1.7**	–	–	**82.7** ± **2.1**	–	–	**84.2** ± **2.3**	–	–
EEGNet	81.5 ± 2.1	1.0000	≈	84.3 ± 2.2	0.7734	≈	83.5 ± 3.6	1.0000	≈
ShallowConvNet	**83.5 ± 1.7**	0.0684	≈	**88.1 ± 2.9**	0.0293	<	80.6 ± 2.4	0.0820	≈
T-GARNet	77.6 ± 0.5	0.0293	>	77.3 ± 0.8	0.0293	>	85.6 ± 1.1	0.7734	≈
IMC-BGT	66.3 ± 1.2	0.0293	>	68.2 ± 1.6	0.0293	>	74.7 ± 2.7	0.0293	>
MultiStream	58.6 ± 0.3	0.0293	>	58.7 ± 0.3	0.0293	>	**86.1 ± 0.9**	0.5273	≈

*Note:* All values are percentages. The shaded row shows CTE-Net’s own results, which serve as the reference for every comparison; the “vs.” columns therefore contrast each baseline with CTE-Net. The symbols > and < indicate a Holm-adjusted difference across repeated model fits in favor of CTE-Net or the compared model, respectively; ≈ indicates that no difference was detected. Boldface indicates the best score for each metric. These comparisons characterize optimization variability and should not be interpreted as participant-level evidence of model superiority. Primary participant-level comparisons are reported in Table 6.

At the participant level, ShallowConvNet exhibited the strongest overall point-estimate profile, whereas CTE-Net followed closely and achieved the second-highest accuracy, specificity, precision, F1-score, and ROC-AUC. EEGNet also showed a competitive overall performance. In contrast, T-GARNet favored sensitivity at the expense of specificity and precision. This imbalance was more pronounced for IMC-BGT and particularly for MultiStream, indicating a greater tendency to classify participants as ADHD.

The participant-level estimates show that CTE-Net maintained robust and comparatively balanced discrimination when each participant, rather than each EEG window, was treated as the evaluation unit. Its joint sensitivity and specificity indicate that its performance was not driven exclusively by the classification of one diagnostic group. Nevertheless, the confidence intervals in Table 5 quantify uncertainty around each model separately and should not be interpreted as tests of pairwise differences. Formal paired comparisons were therefore conducted using the predictions obtained for the same participants.

**Table 5:** Participant-level classification performance of CTE-Net and the baseline models. Point estimates correspond to the mean participant-level performance across ten random training seeds. The 95% confidence intervals were estimated using 5000 stratified bootstrap resamples with the participant as the resampling unit.

Model	Overall		Class-Specific			Ranking
	Accuracy	F1-Score	Sensitivity	Specificity	Precision	ROC-AUC
**CTE-Net**	83.4 (78.2–88.2)	83.8 (78.7–88.4)	86.0 (79.0–92.0)	80.8 (73.2–87.7)	81.8 (76.1–87.6)	90.2 (85.1–94.6)
EEGNet	82.7 (77.7–87.3)	83.4 (78.6–87.8)	87.2 (80.5–92.7)	78.2 (70.3–85.2)	80.1 (74.5–85.8)	89.2 (83.5–93.9)
ShallowConvNet	**84.7** (79.2–89.8)	**84.4** (78.3–89.7)	82.5 (74.0–90.2)	**87.0** (80.0–92.8)	**86.5** (80.5–92.3)	**91.6** (86.6–95.7)
T-GARNet	79.4 (72.5–85.8)	81.4 (75.6–87.0)	90.3 (82.5–96.8)	68.5 (57.0–79.3)	74.2 (67.6–81.4)	85.8 (78.2–92.5)
IMC-BGT	72.7 (66.3–78.8)	75.8 (70.1–81.0)	85.3 (77.0–92.3)	60.0 (50.0–70.0)	68.3 (62.7–74.4)	78.6 (70.2–86.2)
MultiStream	56.2 (51.2–61.4)	68.2 (64.8–71.4)	**93.7** (88.3–98.0)	18.8 (10.2–28.3)	53.6 (50.6–56.8)	60.3 (50.9–69.6)

*Note:* All values are percentages; 95% confidence intervals are given in parentheses. For each participant and random seed, window-level ADHD probabilities were averaged and a threshold of 0.5 was applied to obtain the participant-level decision. Confidence intervals were calculated with the participant as the resampling unit and quantify uncertainty around each model separately; they should not be interpreted as tests of pairwise differences, which are reported in Table 6. The ten seeds represent repeated model fits and were not treated as independent clinical observations. Seed-level mean and standard deviation are reported separately in Table 4 as descriptive measures of optimization variability. CTE-Net, the proposed model, is listed first and set in bold; boldface indicates the best score for each metric.

**Table 6:** Exact participant-level McNemar comparisons between CTE-Net and the baseline models. Consensus decisions were obtained by averaging each participant’s out-of-fold probabilities across the ten random training seeds and applying a threshold of 0.5.

Baseline	Discordant Participants		Exact McNemar Test		vs. CTE-Net
	$n_{10}$	$n_{01}$	$p_{\mathrm{exact}}$	$p_{\mathrm{Holm}}$	
EEGNet	8	7	1.0000	1.0000	≈
ShallowConvNet	12	9	0.6636	1.0000	≈
T-GARNet	12	3	0.0352	0.1055	≈
IMC-BGT	21	7	0.0125	0.0502	≈
MultiStream	47	9	$2.57\times 10^{-7}$	$1.29\times 10^{-6}$	>

*Note: *$n_{10}$ denotes participants correctly classified by CTE-Net but incorrectly classified by the corresponding baseline, whereas $n_{01}$ denotes participants incorrectly classified by CTE-Net but correctly classified by the baseline. Boldface indicates the smallest *p*-value for the McNemar tests. Exact McNemar *p*-values were adjusted across the five comparisons using the Holm procedure. The symbol > indicates a statistically significant difference favoring CTE-Net, whereas ≈ indicates that no significant difference was found after Holm correction.

To determine whether the descriptive performance differences translated into different classification outcomes for the same participants, one consensus decision was obtained for each participant and model by averaging the corresponding out-of-fold probabilities across the ten random seeds and applying the 0.5 threshold. CTE-Net was then compared with each baseline using the exact McNemar test, with Holm correction across the five comparisons. Unlike the exploratory Wilcoxon analysis, this paired analysis used participants, rather than random seeds, as the units of comparison.

As shown in Table 6, the exact participant-level McNemar tests found no statistically significant differences between CTE-Net and EEGNet, ShallowConvNet, T-GARNet, or IMC-BGT after Holm correction. Although the unadjusted comparisons with T-GARNet and IMC-BGT reached the conventional significance threshold, these differences did not remain significant after correction for multiple comparisons. In contrast, CTE-Net correctly classified significantly more participants than MultiStream after Holm correction.

The seed-level and participant-level analyses provide complementary evidence: the Wilcoxon results identify performance differences that were consistently reproduced across repeated model fits using the fixed participant partitions, whereas the McNemar results determine whether those differences translated into a significantly different number of correctly classified participants. Therefore, a difference may be stable across repeated model fits without reaching statistical significance at the participant level after controlling for multiple comparisons.

Taken together, the results indicate that CTE-Net does not uniformly outperform every strong classification baseline, but achieves competitive and comparatively balanced participant-level performance. No significant participant-level differences were detected between CTE-Net and EEGNet, ShallowConvNet, T-GARNet, or IMC-BGT, while a significant advantage over MultiStream was observed. Importantly, CTE-Net complements this competitive predictive performance with an explicit representation of nonlinear, directed, and delayed interchannel dependencies, enabling the representation-level and connectivity analyses presented in the following sections.

### 3.2. Model Robustness and Sensitivity Analyses

To assess the robustness of the principal classification findings, a post hoc signal-quality analysis identified 358 of 8213 EEG windows (4.36%) as potentially contaminated under the primary robust threshold. Excluding these windows produced changes of no more than 0.51 percentage points in the window-level metrics and 0.17 percentage points in the participant-level metrics. Participant-level accuracy changed from 83.42% to 83.50%, while ROC-AUC changed from 90.24% to 90.15%. Moreover, the participant-level proportion of flagged windows was not significantly associated with the consensus ADHD probability across the complete cohort (ρ=0.062, p=0.503). These findings indicate that the principal results were stable after excluding windows with extreme signal-quality indicators. Detailed results are provided in Supplementary Data S2.

Frequency filtering and quality screening, while retaining all participants, substantially reduced participant-level accuracy from 83.5% to 73.9% and ROC-AUC from 90.1% to 81.9%. In contrast, common average reference preserved classification performance but produced low agreement between the learned connectivity patterns (ρ=0.070; Jaccard =0.067). These results indicate that CTE-Net was sensitive to the combined cleaning procedure and that its detailed connectivity patterns depended on reference choice. Complete results are provided in Supplementary Data S4.

Sensitivity to the architectural components was subsequently examined through the component-wise ablation analysis reported in Table 7. The complete CTE-Net architecture achieved the highest point estimates across accuracy, sensitivity, and precision. Removing the Transformer produced the largest reductions across all three metrics. In the exploratory seed-level analysis, these differences remained significant after Holm correction for all three metrics ($p_{\mathrm{Holm}}=0.0117$). This result was also reflected at the participant level, where the exact McNemar comparison significantly favored the complete architecture. Replacing the Transfer Entropy module with temporal mean pooling also reduced the three point estimates; however, the seed-level difference was significant only for accuracy ($p_{\mathrm{Holm}}=0.0410$), and no significant participant-level difference was found. Therefore, the Transformer provides a clear predictive contribution, whereas the evidence for the predictive contribution of the Transfer Entropy module is more moderate. Nevertheless, the latter provides the explicit representation of nonlinear, delayed, and directed interchannel dependencies that distinguishes CTE-Net from the pooling-based variant.

The contribution of the Transfer Entropy module should not be interpreted solely in terms of predictive performance. Replacing it with temporal mean pooling removes the explicit representation of nonlinear, time-delayed, and directional interchannel relationships. Although the participant-level predictive difference between the complete architecture and the pooling-based variant was not statistically significant, CTE-Net preserved an explicit and inspectable directed-connectivity space. This contribution is further supported by the subsequent representation analyses, in which the TE stage produced a more compact within-subject organization, remained consistent across alternative PCA dimensionalities and distance definitions, and enabled the inspection of subject-specific directed-connectivity patterns.

Finally, a post hoc structural sensitivity analysis showed that mean validation accuracy across the 11 configurations ranged from 81.63% to 89.78%, with a median of 86.20%, indicating sensitivity to the joint selection of $D_{x}$, $D_{y}$, τ, and μ. Although several post hoc alternatives exceeded the originally selected configuration in validation, they were not evaluated under the repeated test protocol. Moreover, because the parameters were varied jointly, these results neither identify individual effects nor establish superior generalization. Complete results are provided in Supplementary Data S3.

### 3.3. Evolution and Diagnostic Organization of the Learned Feature Space

**Figure 2:**
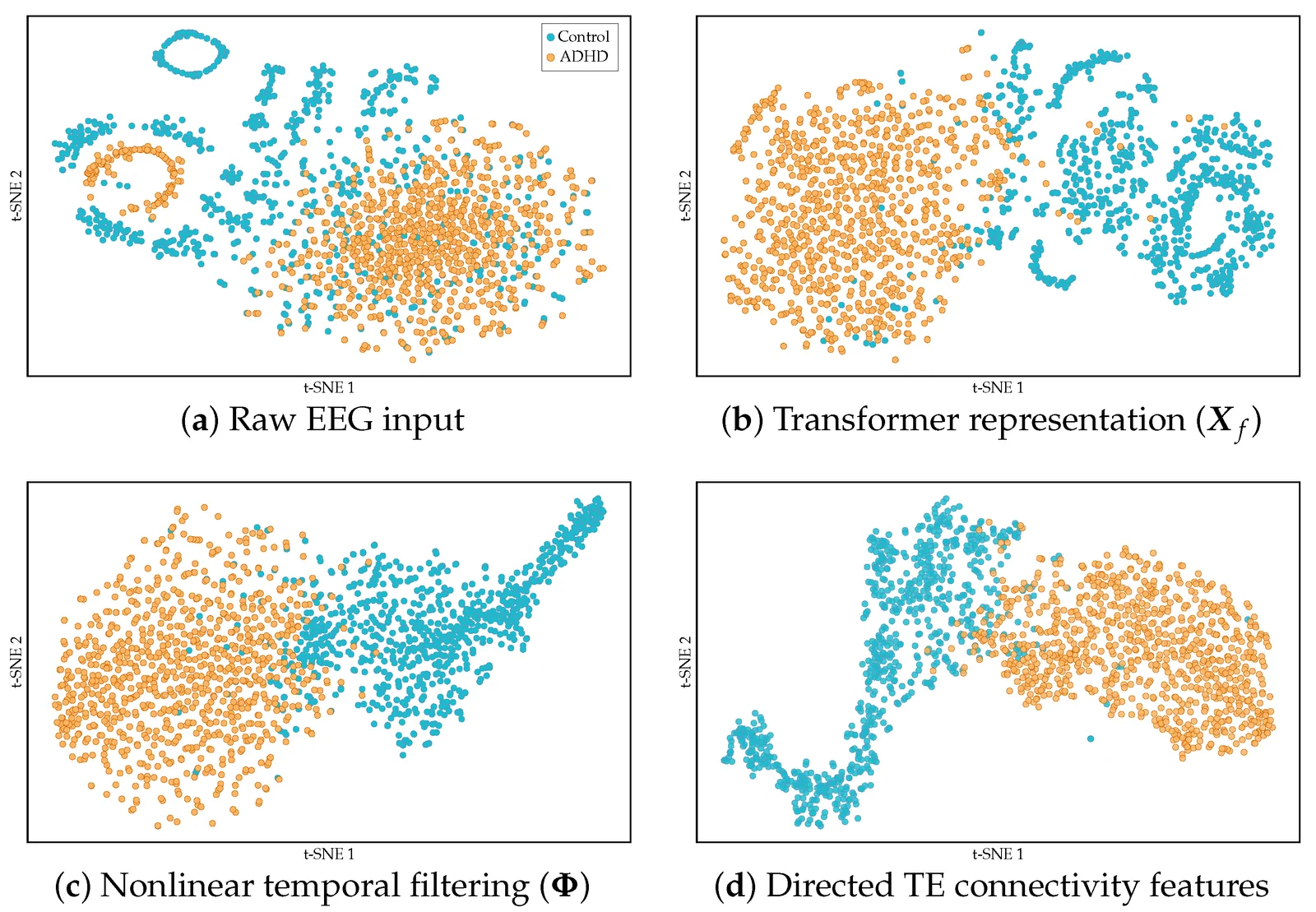
Evolution of the EEG feature space across the main stages of CTE-Net. The t-SNE projections correspond to the raw EEG input, Transformer-contextualized channel representations, nonlinear temporal filtering, and directed Transfer Entropy connectivity features.

Figure 2 qualitatively illustrates the progressive reorganization of the EEG representation across CTE-Net. The raw EEG windows exhibit substantial overlap between ADHD and control participants. Transformer contextualization produces a more structured distribution, although local overlap and participant-dependent subclusters remain. This organization becomes progressively clearer after nonlinear temporal filtering and is most pronounced in the directed Transfer Entropy representation, which shows the most favorable visual arrangement of the two diagnostic groups.

Because a separate t-SNE projection was fitted to each representation, the axes, scales, and absolute distances are not directly comparable across panels. Therefore, Figure 2 is interpreted exclusively as qualitative evidence, whereas diagnostic organization was quantified in the standardized PCA space using the metrics reported in Table 8.

The quantitative results in Table 8 corroborate the progressive organization observed in the t-SNE projections. The raw EEG space showed weak diagnostic structure, with participant centroids from the same class being nearly as distant as those from different classes. Transformer contextualization improved this organization primarily at the participant level, despite the remaining overlap among individual windows. Nonlinear temporal filtering further enhanced both window- and centroid-level organization.

The Transfer Entropy representation achieved the most favorable result for all three metrics. Although some ADHD–control overlap remained, the simultaneous increase in both silhouette coefficients and reduction in the centroid-distance ratio indicate a more coherent diagnostic organization. Differences among stages were significant for all metrics (Friedman tests, $p\leq 3\times 10^{-6}$), and the comparisons between the raw EEG and Transfer Entropy spaces remained significant after Holm correction ($p_{\mathrm{Holm}}=0.0117$). Therefore, the qualitative t-SNE pattern is supported by quantitative evidence computed in the standardized PCA space and does not depend exclusively on the two-dimensional visualization. These findings also indicate that the subsequent reduction in within-subject dispersion was not achieved at the expense of ADHD–control organization.

### 3.4. Reduction of Within-Subject Variability Across CTE-Net Representations

**Figure 3:**
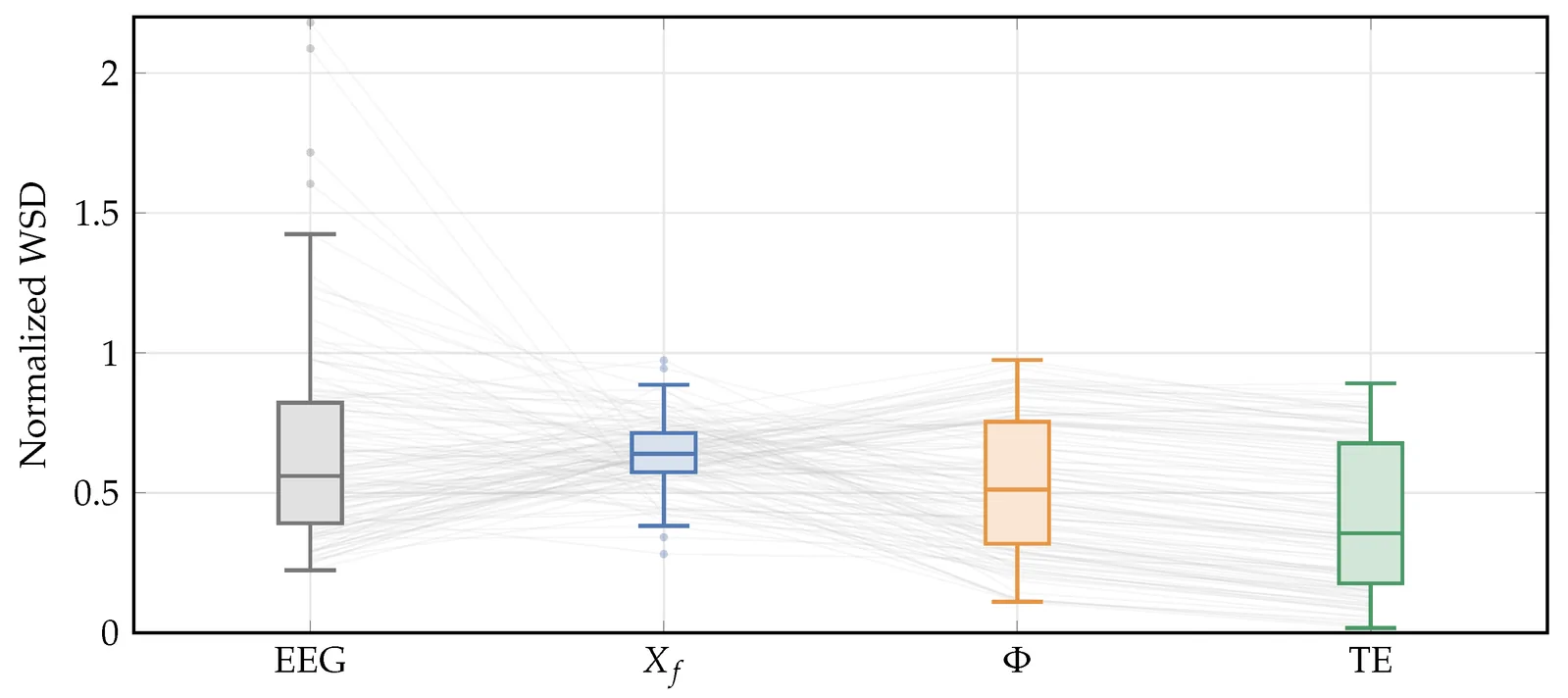
Within-subject dispersion across the four representation stages of CTE-Net. Boxplots summarize the normalized subject-level dispersion after averaging across ten random seeds, while the faint trajectories connect the four representations of each participant. Lower values indicate a more compact organization of EEG windows within the same participant. The directed Transfer Entropy representation exhibited the lowest overall within-subject dispersion.

Figure 3 shows the evolution of normalized within-subject dispersion across the four representation stages. The raw EEG representation exhibited substantial between-participant heterogeneity, including several subjects with markedly elevated dispersion. Transformer contextualization produced a narrower distribution across participants, but did not reduce the median dispersion relative to raw EEG. In contrast, nonlinear temporal filtering initiated a reduction, which became most pronounced after the directed Transfer Entropy transformation.

The Transfer Entropy representation exhibited the lowest median and mean within-subject dispersion among all stages. Relative to raw EEG, the median reduction was 38.35%, with lower dispersion observed in 76 of the 120 participants. This reduction was statistically supported by the Holm-corrected Wilcoxon comparison ($p_{\mathrm{Holm}}=1.09\times 10^{-4}$), while the overall differences among representation stages were significant according to the Friedman test ($\chi^{2}\left(3\right)=69.11$, $p=6.62\times 10^{-15}$). Although individual trajectories were heterogeneous and the reduction was not observed for every participant, the overall pattern indicates that directed-connectivity estimation produces a more compact organization of windows belonging to the same child.

A reduction in within-subject dispersion would not be meaningful if it resulted from a general collapse of the representation space. Two complementary findings argue against a simple global collapse. First, the dispersion measure is normalized by the mean pairwise distance across the complete test set within each representation; therefore, a uniform contraction would reduce both the within-subject and reference distances proportionally and would not, by itself, explain the observed change. Second, the Transfer Entropy stage simultaneously exhibited the most favorable diagnostic organization, including the highest window- and centroid-level silhouette coefficients and the lowest same-class to different-class centroid-distance ratio (Table 8). The cosine-distance analysis without PCA separately confirmed that the reduction in within-subject dispersion was not an artifact of the PCA basis or Euclidean geometry. Together, these findings argue against a simple global feature collapse, although they do not exclude every possible form of representation degeneration.

### 3.5. Robustness to the Comparison Space

**Figure 4:**
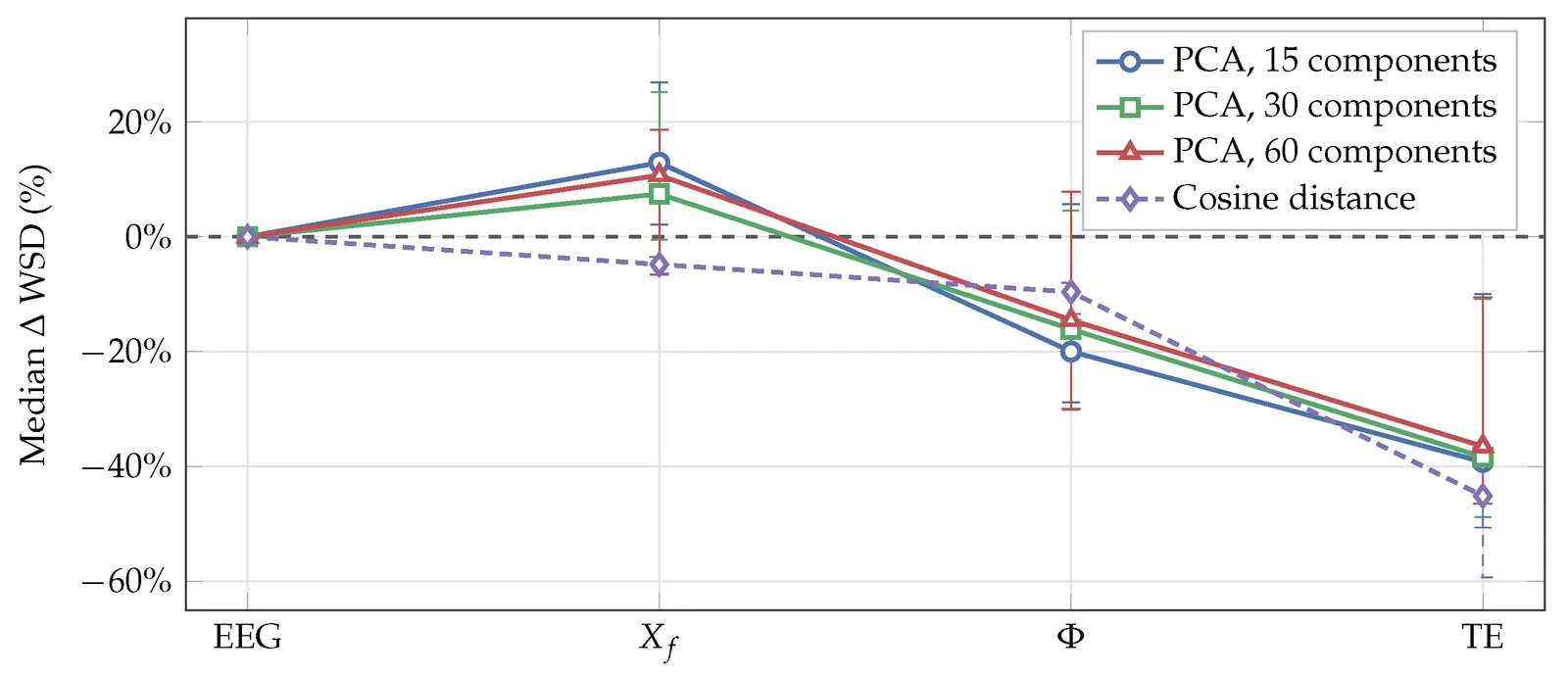
Robustness of within-subject dispersion reduction across alternative comparison spaces. The median percentage change in normalized within-subject dispersion is shown relative to the raw EEG representation for PCA projections with 15, 30, and 60 components, and for pairwise cosine distances computed without PCA. Negative values indicate a reduction in within-subject dispersion, while the vertical bars represent 95% bootstrap confidence intervals.

To determine whether the observed reduction in within-subject dispersion depended on the dimensionality or geometry selected for the analysis, the representations were evaluated using PCA projections with 15, 30, and 60 components, as well as pairwise cosine distances computed directly from the standardized features without PCA. As shown in Figure 4, the intermediate Transformer representation exhibited a moderate increase in dispersion under the PCA-based configurations, whereas the nonlinear temporal filtering stage produced a consistent reduction relative to the raw EEG representation. Most importantly, the Transfer Entropy representation yielded the largest reduction under all evaluated comparison spaces, with median changes of approximately 37–45%. The similar results obtained with 15, 30, and 60 principal components indicate that the effect is not determined by the specific PCA dimensionality selected for the main analysis.

The preservation of the effect when using cosine distances without PCA further indicates that the reduction is not an artifact introduced by the linear projection or by the Euclidean centroid-based geometry. Although the magnitude of the reduction varied across comparison spaces, its direction remained consistent at the Transfer Entropy stage. This result supports the interpretation that the directed-connectivity transformation produces a more compact organization of the windows belonging to the same participant. Therefore, the main finding of reduced within-subject dispersion is robust to alternative dimensionality-reduction settings and distance definitions, complementing the subject-level Friedman and Wilcoxon analyses reported previously.

### 3.6. Stability of Directed Transfer Entropy Representations

Across the out-of-fold test representations, the off-diagonal Transfer Entropy coefficients ranged from −0.0801 to 0.5156. Negative estimates accounted for 10.06% of the coefficients, and no non-finite values were observed. The negative values were retained rather than clipped because non-negativity is not explicitly imposed by the finite-sample matrix-based estimator and should not be interpreted as negative information transfer.

Across the 120 participants, the mean Spearman correlation between each window-level Transfer Entropy vector and the mean of the participant’s remaining windows was 0.743 (95% CI: 0.733–0.753). The mean Jaccard similarity for the strongest 10% of directed connections was 0.492 (95% CI: 0.474–0.509). These results indicate that the global ranking of directed connections remained comparatively stable across windows, whereas the exact composition of the strongest connections showed greater variability.

### 3.7. Subject-Level Analysis of Directed Connectivity Patterns

**Figure 5:**
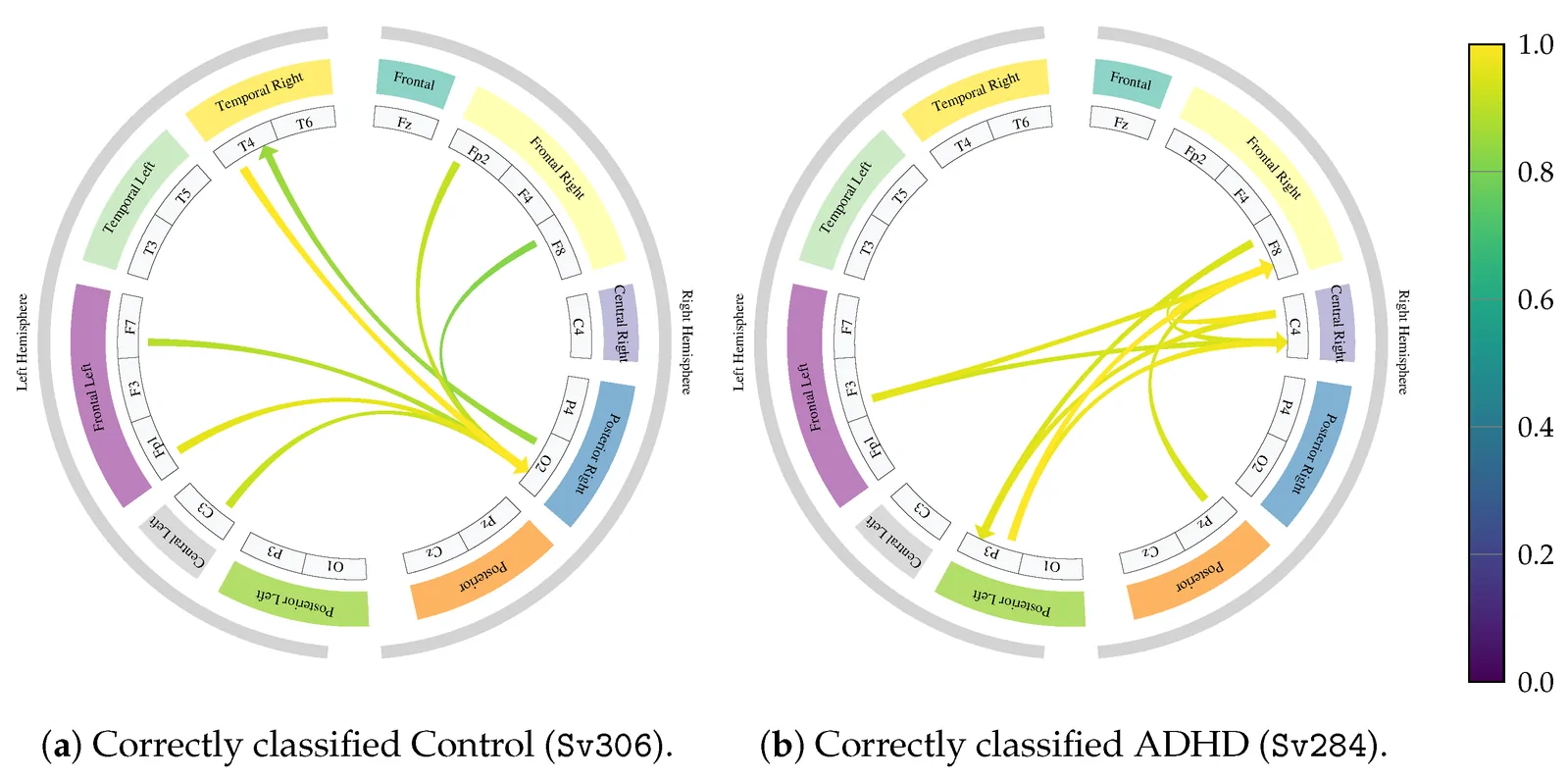
Directed Transfer Entropy connectivity patterns for representative correctly classified subjects. Only the top 5% of the strongest directed connections are shown. Arrow direction indicates the estimated direction of information transfer, while color represents the normalized TE magnitude using a shared range.

To qualitatively illustrate subject-level connectivity patterns associated with correct and incorrect model decisions, four participants were selected from the held-out test partition using their participant-level classification probabilities. These probabilities were obtained by averaging the predicted ADHD probabilities across all windows of each participant. The correctly classified Control and ADHD cases in Figure 5 correspond to the lowest and highest mean ADHD probabilities within their respective classes, whereas the misclassified Control and ADHD cases corresponded to the opposite extremes. The connectivity maps themselves were not used in the selection procedure. For each selected participant, the displayed Transfer Entropy matrix was obtained by averaging the connectivity matrices across all available EEG windows.

Case-level inspection of the correctly classified subjects revealed distinct directed-connectivity organizations. The ADHD subject (v284) exhibited a distributed fronto-centro-parietal pattern involving F8, C4, and P3, with prominent long-range and interhemispheric information-transfer pathways. In contrast, the control subject (v306) showed a more centralized posterior organization, with several relevant pathways converging toward O2. At the organizational level, this contrast is qualitatively consistent with T-GARNet, which reported broader and more diffuse long-range frontoparietal interactions in a correctly classified ADHD case and a more localized pattern in its control counterpart [31]. It also agrees with previous effective-connectivity studies reporting altered directed information transfer in ADHD, particularly across frontal regions and frontal–parietal pathways [7,8]. Nevertheless, these individual patterns should be considered qualitative illustrative cases of model behavior rather than evidence of group-level neurophysiological differences.

**Figure 6:**
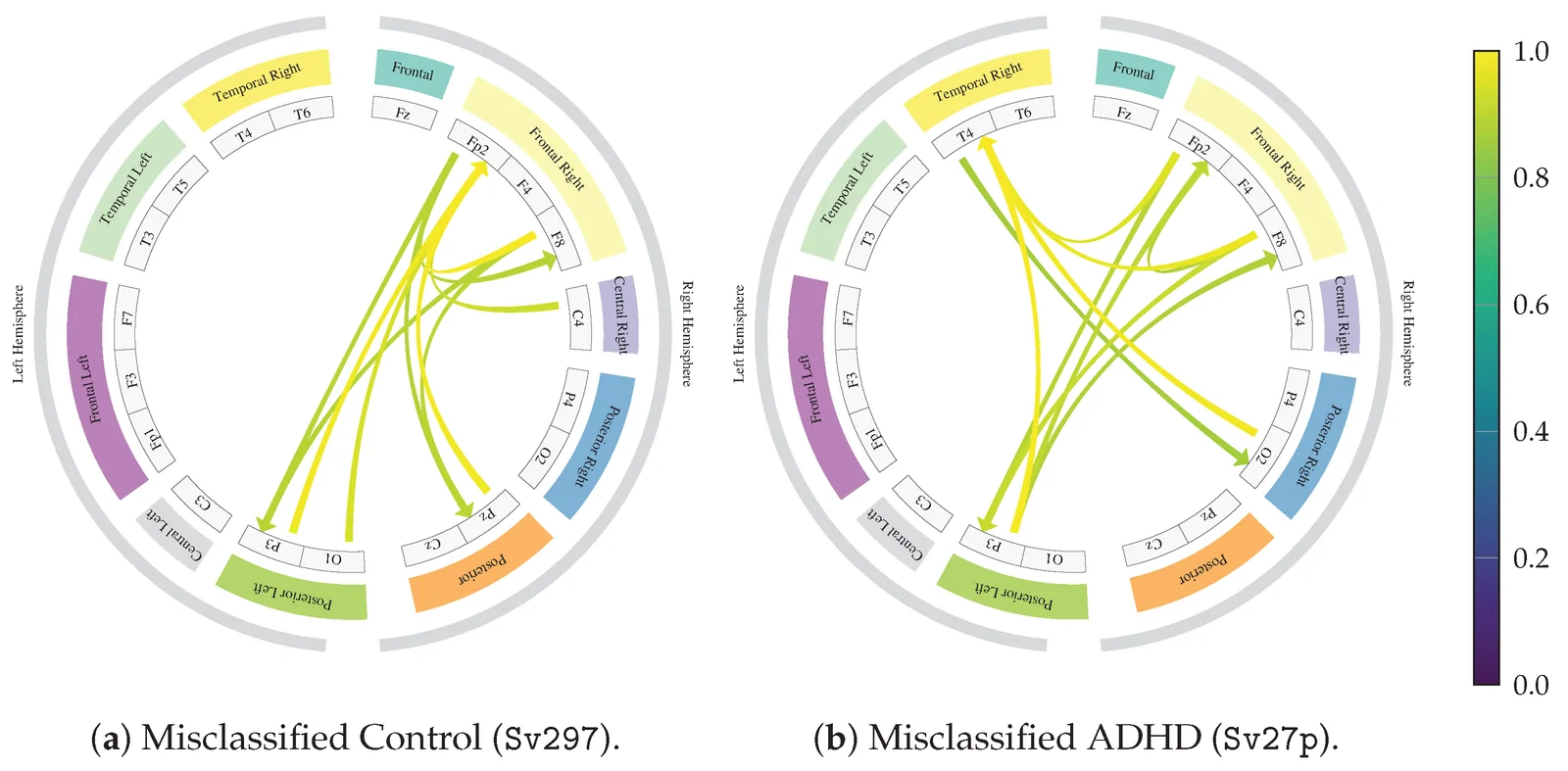
Directed Transfer Entropy connectivity patterns for representative misclassified subjects. Only the top 5% of the strongest directed connections are shown. Arrow direction indicates the estimated direction of information transfer, while color represents the normalized TE magnitude using a shared range.

Consistent with this case-level contrast, the misclassified subjects in Figure 6 exhibited more class-overlapping connectivity organizations. The control subject v297, predicted as ADHD, displayed extended frontoparietal and interhemispheric pathways resembling the distributed pattern of the correctly classified ADHD case, whereas the ADHD subject v27p, predicted as control, showed a less diffuse and less clearly ADHD-related organization. T-GARNet similarly reported mixed connectivity patterns in misclassified cases, with ADHD subjects exhibiting less diffuse networks and controls showing unusually extended long-range interactions [31]. This ambiguity is also compatible with evidence that ADHD affects not only connectivity strength but also the direction of information flow, including disrupted posterior-to-anterior organization during attention tasks [44], suggesting that subject-specific departures from the patterns learned for each class may contribute to classification errors.

## 4. Concluding Remarks and Future Work

This study introduces CTE-Net, an end-to-end architecture for EEG-based ADHD classification that integrates Transformer-based contextualization, nonlinear temporal filtering, Takens delay-coordinate reconstruction, and differentiable kernel Transfer Entropy estimation. The proposed model was designed not only to discriminate between ADHD and control EEG windows, but also to provide an explicit representation of nonlinear, time-delayed, and directed interchannel dependencies. Under a subject-independent evaluation protocol using five fixed stratified group folds and ten random training repetitions, CTE-Net achieved competitive window-level classification performance, with an accuracy of 80.9±1.7%, precision of 82.7±2.1%, and sensitivity of 84.2±2.3%.

At the participant level, CTE-Net achieved an accuracy of 83.4% (95% CI: 78.2–88.2) and an ROC-AUC of 90.2% (95% CI: 85.1–94.6), while maintaining comparatively balanced sensitivity and specificity. The exploratory seed-level Wilcoxon analysis identified performance differences that were consistently reproduced across repeated model fits. However, the primary exact participant-level McNemar tests found no statistically significant differences between CTE-Net and EEGNet, ShallowConvNet, T-GARNet, or IMC-BGT after Holm correction, whereas CTE-Net significantly outperformed MultiStream. These findings indicate that CTE-Net achieves competitive participant-level predictive performance while additionally providing an inspectable, channel-indexed representation of directed predictive dependencies.

Beyond predictive performance, the main contribution of CTE-Net lies in the organization of the learned EEG representation space. The directed Transfer Entropy stage produced the most favorable diagnostic structure, as reflected by increased window- and centroid-level silhouette coefficients and a reduced same-class to different-class centroid-distance ratio. Importantly, this improvement was not accompanied by an increase in within-subject dispersion. On the contrary, the Transfer Entropy representation exhibited the lowest within-subject variability among all analyzed stages, with a median reduction of 38.35% relative to raw EEG and lower dispersion in 76 of the 120 participants. This effect remained consistent across alternative PCA dimensionality settings and cosine-distance analyses, indicating that the compactness of the representation was not an artifact of a particular projection space or distance metric. These findings suggest that directed information-flow representations can attenuate window-specific fluctuations while retaining diagnostically meaningful group structure.

The subject-level connectivity inspection further supports the proposed framework’s potential interpretability. Correctly classified ADHD and control cases showed qualitatively distinct directed-connectivity organizations, with ADHD examples displaying more distributed fronto-centro-parietal and interhemispheric information-transfer patterns. In contrast, control examples tended to exhibit more localized posterior organization. Conversely, misclassified subjects exhibited connectivity patterns that partially resembled those of the opposite class, suggesting that classification errors may arise from subject-specific overlap in the organization of directed information flow. Nevertheless, these case-level analyses should be interpreted as illustrative evidence rather than definitive neurophysiological biomarkers. Overall, CTE-Net provides a compact and interpretable computational framework for modeling directed EEG interactions in pediatric ADHD, complementing conventional deep-learning classifiers with representation-level and connectivity-level analyses. As a single-dataset methodological study, these results should be regarded as proof-of-concept evidence, with external validation and prospective clinical assessment required before any diagnostic or decision-support claims can be established.

Despite the findings achieved, future work should address some identified limitations. First, CTE-Net was evaluated on a single public pediatric EEG dataset comprising 120 participants. The public release does not provide participant-level age and sex metadata linked to the individual EEG recordings. Consequently, their associations with the model predictions or learned representations could not be evaluated, and residual demographic confounding related to these variables cannot be excluded.

Although the dataset reports a history of methylphenidate (Ritalin) use for up to six months, medication status during EEG acquisition was unavailable; therefore, medication-related effects on EEG activity and model outputs cannot be excluded. Hence, new validation studies should include larger, independent, multi-site cohorts with heterogeneous acquisition protocols to assess the generalizability of this kind of model. Such studies should also explicitly model potential confounding factors (e.g., medication status, comorbidities, age, and developmental variability) to determine whether the learned directed-connectivity patterns remain stable across clinically relevant subgroups.

Second, the use of a single hyperparameter configuration selected across the predefined folds represents a methodological limitation. Future work should complement the present evaluation with fully nested cross-validation, in which hyperparameter optimization is performed independently within each outer training partition, together with validation using independent external cohorts. Third, although Transfer Entropy provides a directed measure of predictive information flow, it should not be interpreted as causal connectivity without additional assumptions, experimental controls, or causal modeling frameworks.

Because the analysis was performed at the sensor level, the learned channel-indexed dependencies may also be influenced by volume conduction, spatial source mixing, and the EEG reference. Therefore, they should not be interpreted as direct interactions between anatomically localized cortical sources [45,46]. The filtered and quality-screened analysis reduced performance, whereas common average reference preserved classification but altered the learned connections. These findings indicate sensitivity to preprocessing and reference choice; therefore, residual artifacts cannot be excluded and the connectivity patterns should not be regarded as reference-independent. Because filtering and window exclusion were applied jointly, their individual effects could not be isolated.

An additional architectural consideration is that the Transformer encoder does not incorporate explicit positional encoding. Consequently, the self-attention mechanism does not, by itself, encode temporal order or temporal distance based on token position. Instead, temporal ordering and delayed dependencies are explicitly modeled in the subsequent channel-wise temporal-filtering and Takens delay-coordinate reconstruction stages. Future work should assess whether incorporating absolute or relative positional representations [28,47] provides additional benefits for global contextualization and downstream directed-connectivity estimation.

Future methodological extensions may incorporate frequency-specific or multiband Transfer Entropy modules, multivariate or conditional TE formulations to reduce indirect pairwise effects, and graph-based neural architectures or sparsity-inducing regularization to obtain more structured and neurophysiologically constrained connectivity representations. Finally, future work should expand interpretability through group-level statistical analysis of directed connections, stability assessment of subject-specific TE patterns, and integration with clinical and behavioral variables, with the long-term goal of developing EEG-based decision-support tools that complement expert clinical assessment of ADHD.

## Figures and Tables

**Table 1:** Main characteristics of the EEG dataset and the cohort considered in the present study.

Characteristic	ADHD Group	Control Group
*Cohort and demographics*		
Participants, *n*	60	60
Sex, boys/girls	48/12	50/10
Age, years (mean ± SD)	9.62±1.75	9.85±1.77
Age range, years	7–12	
Group definition	DSM-IV diagnostic criteria	Typically developing
Medication history	Methylphenidate for no more than six months	Not reported
*EEG acquisition*		
Channels	19, international 10–20 system	
Electrodes	Fz, Cz, Pz, C3, T3, C4, T4, Fp1, Fp2, F3, F4, F7, F8, P3, P4, T5, T6, O1, O2	
Reference	A1 and A2 earlobe electrodes	
Sampling rate	128 Hz	
*Experimental protocol*		
Task	Visual continuous-performance task based on cartoon images	
Stimuli	Between 5 and 16 characters per image	
Recording duration	Determined by the response speed of each participant	
*Data availability*		
Source	Public ADHD EEG dataset available through IEEE DataPort [33]	

*Note:* The original dataset comprised 121 children. One ADHD participant was excluded before any analysis to define the balanced cohort of 120 participants reported here. Characteristics that apply to both groups span the two group columns.

**Table 2:** Compact CTE-Net architecture for EEG-based ADHD classification.

Layer	Variable	Output Dimension	Configuration
**Block 1: Contextualization**			
Input EEG	X	C×T	C=19,T=512
Transpose	${\text{X}}^{⊤}$	T×C	–
Input projection	E	T×d	d=128
Transformer encoder	H	T×d	$L=1,\;n_{h}=1,\;d_{h}=128,\;d_{ff}=128,\;p=0.5$
Channel projection	${\text{X}}_{f}^{⊤}$	T×C	d→C
Transpose	${\text{X}}_{f}$	C×T	–
**Block 2: Transfer Entropy**			
Temporal filtering	Φ	$C\times T_{\phi }$	$K=83,\;T_{\phi }=107,\;s=1,\;\mathrm{pool}=4$
Takens source state	$Z_{x^{-}}$	$C\times T_{z}\times D_{x}$	$D_{x}=6,\;T_{z}=48$
Takens target past	$Z_{y^{-}}$	$C\times T_{z}\times D_{y}$	$D_{y}=1,\;T_{z}=48$
Takens target present	$z_{y}$	$C\times T_{z}\times 1$	$T_{z}=48$
Kernel matrices	$K_{x^{-}},\;K_{y^{-}},\;K_{y}$	19×48×48	$\tau =2,\;\mu =2,\;a=1,\;\ell =1,\;\alpha_{\mathrm{RQ}}=1$
Transfer Entropy	TE	C×C	$\alpha_{R}=2$
Flatten	${\text{h}}_{TE}$	C(C−1)	–
**Block 3: Classification**			
Dense	h	*H*	H=64
Dropout	–	64	p=0.5
Output layer	$\hat{y}$	*O*	O=1

**Table 3:** Window-level classification performance of CTE-Net and the baseline models. Results are reported as the mean and sample standard deviation across ten random training seeds after averaging the five test folds within each seed.

Model	Overall		Class-Specific				Ranking
	Accuracy	BAcc	Sens.	Spec.	Prec.	F1	ROC-AUC
**CTE-Net**	80.9 ± 1.7	80.6 ± 1.8	84.2 ± 2.3	77.0 ± 3.2	82.7 ± 2.1	82.9 ± 1.5	87.9 ± 1.5
EEGNet	81.5 ± 2.1	81.4 ± 2.1	83.5 ± 3.6	79.3 ± 4.0	84.3 ± 2.2	83.1 ± 2.2	88.8 ± 2.5
ShallowConvNet	**83.5 ± 1.7**	**83.7 ± 1.8**	80.6 ± 2.4	** 86.9 ± 3.4**	** 88.1 ± 2.9**	**83.2 ± 2.0**	**90.4 ± 1.7**
T-GARNet	77.6 ± 0.5	76.7 ± 0.6	85.6 ± 1.1	67.8 ± 1.9	77.3 ± 0.8	80.8 ± 0.4	84.0 ± 0.4
IMC-BGT	66.3 ± 1.2	65.3 ± 1.4	74.7 ± 2.7	55.8 ± 4.5	68.2 ± 1.6	70.8 ± 1.0	71.2 ± 1.0
MultiStream	58.6 ± 0.3	55.2 ± 0.4	**86.1 ± 0.9**	24.4 ± 1.4	58.7 ± 0.3	69.8 ± 0.3	55.3 ± 0.4

*Note:* All values are percentages. ADHD was treated as the positive class, so sensitivity denotes the true-positive rate for ADHD and specificity the true-negative rate for controls. BAcc denotes balanced accuracy. Values were first averaged across the five test folds within each seed and then summarized across the ten random training seeds. The seeds represent repeated model fits and were not treated as independent clinical observations. The shaded row indicates the proposed model, and boldface indicates the best score for each metric.

**Table 7:** Ablation study of CTE-Net. Results are reported as the mean and sample standard deviation across ten random training seeds after averaging the five subject-wise test folds within each seed. The last column reports the primary participant-level exact McNemar comparisons against CTE-Net.

Model Variant	Architecture			Window-Level Performance (%)			Participant Level
	Trans.	TE	Representation	Accuracy	Sensitivity	Precision	$p_{\mathrm{Holm}}$
Without Transformer	×	✔	Rational quadratic	73.4 ± 2.2	78.0 ± 2.8	75.7 ± 2.5	**0.0338**
Without TE module	✔	×	Temporal mean pooling	78.7 ± 0.8	82.3 ± 2.8	80.8 ± 1.6	0.2891
**CTE-Net (proposed)**	✔	✔	Rational quadratic	**80.9 ± 1.7**	**84.2 ± 2.3**	**82.7 ± 2.1**	–

*Note:* All variants were evaluated using the same fixed subject-wise folds and ten random training seeds. The seeds represent repeated model fits and were not treated as independent clinical observations. Participant-level comparisons were performed using one consensus decision per participant and model. Exact McNemar *p*-values were adjusted across the two ablation comparisons using the Holm procedure. Bold *p*-values indicate statistically significant differences favoring CTE-Net after correction, and the shaded row indicates the complete architecture.

**Table 8:** Diagnostic organization across the representation stages of CTE-Net. Values correspond to averages across the ten training repetitions after averaging the five test folds within each repetition.

Representation	Class Silhouette ↑		Same/Different
Stage	Window Level	Centroid Level	Class Ratio ↓
Raw EEG	0.0558	0.0210	0.9887
Transformer ($X_{f}$)	0.0712	0.2640	0.7056
Temporal filter (Φ)	0.1326	0.3316	0.6326
Transfer Entropy	**0.2148**	**0.3557**	**0.6031**

*Note:* Arrows indicate the direction of more favorable diagnostic organization: higher silhouette coefficients (↑) and lower same-to-different class centroid-distance ratios (↓). All quantities were computed in the standardized PCA space described in Section 2.4. Bold values indicate the most favorable value for each measure, obtained in every case at the directed Transfer Entropy stage.

## Data Availability

The EEG dataset used in this study is publicly available at https://ieee-dataport.org/open-access/eeg-data-adhd-control-children (accessed on 1 July 2025). The source code required to reproduce the procedures described in this work is available in the project repository: https://github.com/alegomezri/CTE-Net (accessed on 1 July 2025).

## Supplementary Materials

The following supporting information can be downloaded at https://www.mdpi.com/article/10.3390/s26185786/s1: Supplementary Data S1: Row-normalized window-level confusion matrices for CTE-Net and all baseline models (CSV); Supplementary Data S2: Signal-quality thresholds, flagged-window summaries, artifact indicators, participant coverage, and window- and participant-level sensitivity results (CSV); Supplementary Data S3: Post hoc joint structural sensitivity results for the Transfer Entropy parameters, including configuration-level validation accuracies and summary statistics (CSV); Supplementary Data S4: Complete preprocessing and reference sensitivity analyses for the frequency-filtered and quality-screened condition and the common-average-reference condition. The files include window- and participant-level metrics, paired bootstrap differences, prediction agreement, exact McNemar comparisons, connectivity concordance results, and the corresponding analysis code.
